# Human Factors, Human-Centered Design, and Usability of Sensor-Based Digital Health Technologies: Scoping Review

**DOI:** 10.2196/57628

**Published:** 2024-11-15

**Authors:** Animesh Tandon, Bryan Cobb, Jacob Centra, Elena Izmailova, Nikolay V Manyakov, Samantha McClenahan, Smit Patel, Emre Sezgin, Srinivasan Vairavan, Bernard Vrijens, Jessie P Bakker, Digital Health Measurement Collaborative Community (DATAcc) hosted by DiMe

**Affiliations:** 1 Division of Cardiology and Cardiovascular Medicine Department of Heart, Vascular, and Thoracic Children’s Institute, Cleveland Clinic Children’s Cleveland, OH United States; 2 Cleveland Clinic Children’s Center for Artificial Intelligence Department of Heart, Vascular, and Thoracic Children’s Institute, Cleveland Clinic Children’s Cleveland, OH United States; 3 Department of Pediatrics Cleveland Clinic Lerner College of Medicine of Case Western Reserve University Cleveland, OH United States; 4 Healthcare Innovations Delivery, Neurology, Medical Affairs Genentech San Francisco, CA United States; 5 Digital Medicine Society Boston, MA United States; 6 Koneksa Health New York, NY United States; 7 Data Science and Digital Health Johnson & Johnson Innovative Medicine Beerse Belgium; 8 The Abigail Wexner Research Institute Nationwide Children’s Hospital Columbus, OH United States; 9 Johnson & Johnson Innovative Medicine New Jersey, NJ United States; 10 AARDEX Group Liège Belgium; 11 Division of Sleep and Circadian Disorders Mass General Brigham Boston, MA United States; 12 Division of Sleep Medicine Harvard Medical School Boston, MA United States; 13 See Acknowledgements

**Keywords:** digital health, remote, decentralized, sensors, connected care, usability, ergonomics, human-centered design, user experience, systematic scoping review, human factors, screening, clinicians, wearable, mobile phone

## Abstract

**Background:**

Increasing adoption of sensor-based digital health technologies (sDHTs) in recent years has cast light on the many challenges in implementing these tools into clinical trials and patient care at scale across diverse patient populations; however, the methodological approaches taken toward sDHT usability evaluation have varied markedly.

**Objective:**

This review aims to explore the current landscape of studies reporting data related to sDHT human factors, human-centered design, and usability, to inform our concurrent work on developing an evaluation framework for sDHT usability.

**Methods:**

We conducted a scoping review of studies published between 2013 and 2023 and indexed in PubMed, in which data related to sDHT human factors, human-centered design, and usability were reported. Following a systematic screening process, we extracted the study design, participant sample, the sDHT or sDHTs used, the methods of data capture, and the types of usability-related data captured.

**Results:**

Our literature search returned 442 papers, of which 85 papers were found to be eligible and 83 papers were available for data extraction and not under embargo. In total, 164 sDHTs were evaluated; 141 (86%) sDHTs were wearable tools while the remaining 23 (14%) sDHTs were ambient tools. The majority of studies (55/83, 66%) reported summative evaluations of final-design sDHTs. Almost all studies (82/83, 99%) captured data from targeted end users, but only 18 (22%) out of 83 studies captured data from additional users such as care partners or clinicians. User satisfaction and ease of use were evaluated for 83% (136/164) and 91% (150/164) of sDHTs, respectively; however, learnability, efficiency, and memorability were reported for only 11 (7%), 4 (2%), and 2 (1%) out of 164 sDHTs, respectively. A total of 14 (9%) out of 164 sDHTs were evaluated according to the extent to which users were able to understand the clinical data or other information presented to them (understandability) or the actions or tasks they should complete in response (actionability). Notable gaps in reporting included the absence of a sample size rationale (reported for 21/83, 25% of all studies and 17/55, 31% of summative studies) and incomplete sociodemographic descriptive data (complete age, sex/gender, and race/ethnicity reported for 14/83, 17% of studies).

**Conclusions:**

Based on our findings, we suggest four actionable recommendations for future studies that will help to advance the implementation of sDHTs: (1) consider an in-depth assessment of technology usability beyond user satisfaction and ease of use, (2) expand recruitment to include important user groups such as clinicians and care partners, (3) report the rationale for key study design considerations including the sample size, and (4) provide rich descriptive statistics regarding the study sample to allow a complete understanding of generalizability to other patient populations and contexts of use.

## Introduction

Sensor-based digital health technologies (sDHTs), defined as connected digital medicine products that process data captured by mobile sensors using algorithms to generate measures of behavioral and/or physiological function [1], have been increasingly adopted in both research and health care in recent years [2,3]. sDHTs include products designed to capture data passively (such as continuous glucose monitors and wearables for monitoring sleep) or during active tasks (such as mobile spirometry or smartphone-based cognitive assessments) from wearable, implantable, ingestible, or ambient tools. Implementation of sDHTs requires interactions across the hardware containing the sensor or sensors, the software that is used to convert sensor data to health-related measures, and the users (who could be consumers, patients, clinicians, and more) who interact at one or more stages of data capture. Given this complexity and the increasing use of sDHTs, defining and understanding best practices for human factors, human-centered design, and usability (defined in Textbox 1) of sDHTs is a critical need. Although regulatory guidance focused on the usability of medical devices is well established, sDHTs require unique consideration because (1) sDHTs used in clinical research studies for data capture may or may not be regulated medical devices [4], (2) research participants likely have different motivations and needs related to their use of the technology, (3) sDHTs are often used over much longer time periods in research compared with health care settings, and (4) digital measures captured in large studies may be analyzed with limited human oversight or clinical interpretation.

The methodological approaches taken toward sDHT usability evaluation have varied substantially [5,6], casting light on the many challenges in implementing these tools into clinical trials and patient care at scale across diverse patient populations [7,8]. For example, some studies have adopted questionnaires developed for products and systems other than sDHTs [9], while others have described the approach to participatory design alongside qualitative data capture [10]. Inadequate attention to human-centered design and usability testing approaches can hinder the evaluation of health care interventions, contribute to insufficient adoption, perpetuate health disparities, increase costs, and potentially introduce safety risks [11-14]. Thus, integrating human factors considerations in the design, development, and evaluation of sDHTs is critical to improving their likelihood of being adopted and properly utilized in a way that is safe, effective, inclusive, and optimizes the user experience.

While several systematic reviews have focused on understanding and quantifying the usability of digital health products for specific applications [15-19], their focus has primarily been on study outcomes rather than evaluating methodological approaches. Recognizing the urgency of addressing sDHT usability-related challenges, a precompetitive collaboration within the Digital Health Measurement Collaborative Community (DATAcc) hosted by the Digital Medicine Society (DiMe) undertook a scoping review to highlight studies that have performed a usability-related evaluation for sDHTs, outline the dimensions of usability data that were assessed, and highlight the methods of usability evaluation. Our objective was to explore the current landscape and identify gaps, which will inform the development and dissemination of recommendations and an evidence-driven evaluation framework of sDHTs as being fit for purpose from a usability perspective.

**Definitions.**

**Human factors**

- The application of knowledge about human behavior, abilities, limitations, and other characteristics of users to the design and development of a sensor-based digital health technology (sDHT) to optimize usability within a defined intended use or context of use. This definition incorporates terminology and concepts from the US Food and Drug Administration (FDA) [20], the UK Medicines and Health Care Products Regulatory Agency (MHRA) [21], and the National Medical Products Administration (NMPA) of China (translated) [22].

**Human-centered design**

- An approach to interactive systems that aims to make systems usable and useful by focusing on the users, their needs and requirements, and by applying human factors and usability knowledge and techniques, as defined in the International Organization for Standardization (ISO) 9241-210:2019 standard [23].

**Usability**

- The extent to which an sDHT can be used to achieve specified goals with ease, efficiency, and user satisfaction within a defined intended use or context of use. This definition incorporates terminology and concepts from the FDA [20], the MHRA [21], the NMPA (translated) [22], and ISO 9241-210:2019 [23].

## Methods

### Overview

We followed the PRISMA (Preferred Reporting Items for Systematic Reviews and Meta-Analyses) guidelines for scoping reviews (Multimedia Appendix 1) [24]. As a scoping review, this work did not meet the criteria for registration on PROSPERO [25]. The protocol is available from the corresponding author.

### Literature Search

We completed our literature search in PubMed using search terms designed in 6 layers as follows (terms within each layer were separated by the Boolean operator “OR”, while the layers themselves were separated using “AND” or “NOT”): (1) Medical Subject Heading (MeSH; [26]) term for human participants; (2) MeSH terms related to sDHTs, such as wearable electronic devices and digital technology; (3) keywords related to sDHTs such as wear* (asterisk indicates truncation), remote, and connected; (4) keywords related to human-centered design, usability, human factors, and ergonomics; (5) exclusion of out-of-scope publication types such as editorials and case reports; and (6) published between January 1, 2013, and May 30, 2023. The complete search string is provided in Table S1 in Multimedia Appendix 2.

To avoid potentially overlooking novel or emerging technologies, the search terms did not include descriptions of specific sensor types (such as accelerometer), form factors (such as watch), methodology (such as actigraphy), wear location (such as wrist), or technology make or model.

### Study Selection

We systematically screened publications identified in the literature search based on the PICO (patients/participants; intervention; comparator; outcomes) eligibility criteria outlined in Table 1, designed to identify studies describing the incorporation of knowledge about human behavior, abilities, limitations, and other characteristics of users to the design and development process; human-centered design; and ease of use, efficiency, or user satisfaction of sDHTs. Studies reporting sDHT adherence (eg, average wear time) or measurement success metrics (eg, percentage of in-range measurements obtained) were considered out of scope unless they reported one of the aforementioned concepts.

Two independent investigators (JC and JPB) began by screening a random selection of 20% of publications; disagreements were resolved by consensus, and clarifications were made to the wording of the eligibility criteria to reduce ambiguity. The same two investigators then reviewed another random selection of 20% of publications; it was determined a priori that if the reviewers were in agreement for ≥90% of these publications, the remaining 60% would be reviewed by a single investigator (JC) as described elsewhere [27,28].

**Table 1:** Study selection eligibility criteria.

PICO^a^ framework^b^	Eligibility criteria
Patient or participant	Exclude studies that do not report data collected from human participants
Intervention	Exclude studies that do not assess a specific sDHT^c^, defined according to the definition of BioMeT in the V3 framework^d^: Connected Interpreted as a digital method of data transfer from the sDHT to the location of data analysis, either wired or wireless Mobile Interpreted as the tool being capable of collecting data in the out-of-clinic setting, although the study may have deployed the tool in clinic Sensor-based Interpreted as the tool containing at least one sensor sampling a physical construct such as acceleration, light, or temperature Used for purposes of measurement, diagnosis, and/or treatment of a behavioral or physiological function
Comparator	N/A^e^
Outcome or outcomes	Exclude studies that do not report data on human factors, human-centered design, or usability (see Textbox 1 for definitions)

^a^PICO: patients/participants; intervention; comparator; outcomes.

^b^The PICO framework is described by Eriksen and Frandsen [29].

^c^sDHT: sensor-based digital health technology.

^d^Throughout this review, we refer to “sensor-based digital health technology” (sDHT); however, this was operationalized according to the definition of “biometric monitoring technology” (BioMeT) as described in Goldsack et al [1].

^e^Not applicable.

### Data Extraction and Analysis

Data extraction fields included study design and sample characteristics; the type, maturity, make or model, form factor, and wear location (if applicable) of each sDHT evaluated along with the health concept or concepts generated by each sDHT; the methodological approaches; and the types of usability-related data reported in each study. Most fields for data capture were categorical, with categories created in advance to minimize error. Extraction from each publication was undertaken by one of three investigators with adjudication by an independent investigator as needed.

Categories of usability-related data are described in Table 2, and compiled based on the literature including the International Organization for Standardization (ISO) 9241-210:2019 standard [23] and Nielsen’s [30] usability attributes, as well as the studies identified in this review; that is, data not clearly fitting into an existing category were extracted and categorized post hoc. We acknowledge that there are various models for capturing data describing usability and related topics [31]; however, there is no single standard that has been widely adopted.

Consistent with the goal of a scoping review, all data were analyzed descriptively.

**Table 2:** Categories of usability-related data extracted from eligible papers.

Category	Definition^a,b^
User satisfaction	The extent to which a user finds the sDHT^c^ to be pleasant to use, which may reflect trust, comfort, aesthetics, engagement, desirability, emotional response, and other considerations. Always captured through self-report.
Ease of use	The ease with which a user is able to perform user tasks. Can be captured through self-report (such as the mental demand or effort required to complete a task) or objective measures (such as the number of actions, number of attempts, or time required to complete a task).
Efficiency	The ease with which a user is able to perform user tasks after having learned how to use the sDHT. Captured according to the definition of ease of use above.
Learnability^d^	The ease with which a user is able to perform user tasks during their first encounter with the sDHT. Captured according to the definition of ease of use above.
Memorability	The ease with which a user is able to perform user tasks after a period of nonuse, assessed in a test-retest paradigm. Captured according to the definition of ease of use above.
Usefulness^e^	The extent to which a user finds the sDHT, or its specific features or functions, to be valuable, productive, or helpful. Always captured through self-report.
Use errors^f^	An action or lack of action that may result in a use-related hazard (a potential source of harm), as well as error recovery defined as the ability of a user to make a correction following a use error in order to complete a task. Can be captured through self-report or objective assessments.
Technical performance or malfunctions	Technical performance, such as page load times, or the number, type, and severity of errors associated with sDHT malfunction. Can be captured through self-report or objective assessments.
Readability	The reading skills a user must possess to understand information presented to them through the sDHT itself, or through written materials such as instructions for use, cautions, warnings, or contraindications; [32]. Always captured through objective assessments, and typically reported as a reading grade.
Understandability or actionability	The extent to which users of diverse backgrounds, languages, and varying levels of health literacy understand (1) the clinical data or other information, such as instructions, cautions, warnings, and contraindications, presented to them; and (2) the actions or tasks they should complete in response, such as an sDHT-derived blood glucose measurement requiring an adjustment to medication [32]. Always captured through objective assessments.

^a^Note that in the definitions, “self-report” includes data captured through surveys, interviews, and focus groups, while “objective” includes data captured through observation (direct or video) or through the sDHT itself (or any related software) such as timestamps, app crash reports, and page load times.

^b^Comfort and trust were extracted separately for the purposes of this review.

^c^sDHT: sensor-based digital health technology.

^d^Learnability refers to the operation of the sDHT rather than a practice effect associated with a research study outcome or endpoint.

^e^We have adopted the term usefulness instead of utility, to avoid confusion with clinical utility, which refers to the extent to which implementing a medical product leads to improved health outcomes or provides useful information about diagnosis, treatment, management, or prevention of disease [33].

^f^Study outcomes that come after “use errors” are not typically considered usability data, but are related concepts often captured during usability evaluations.

## Results

### Literature Search and Study Selection

**Figure 1:**
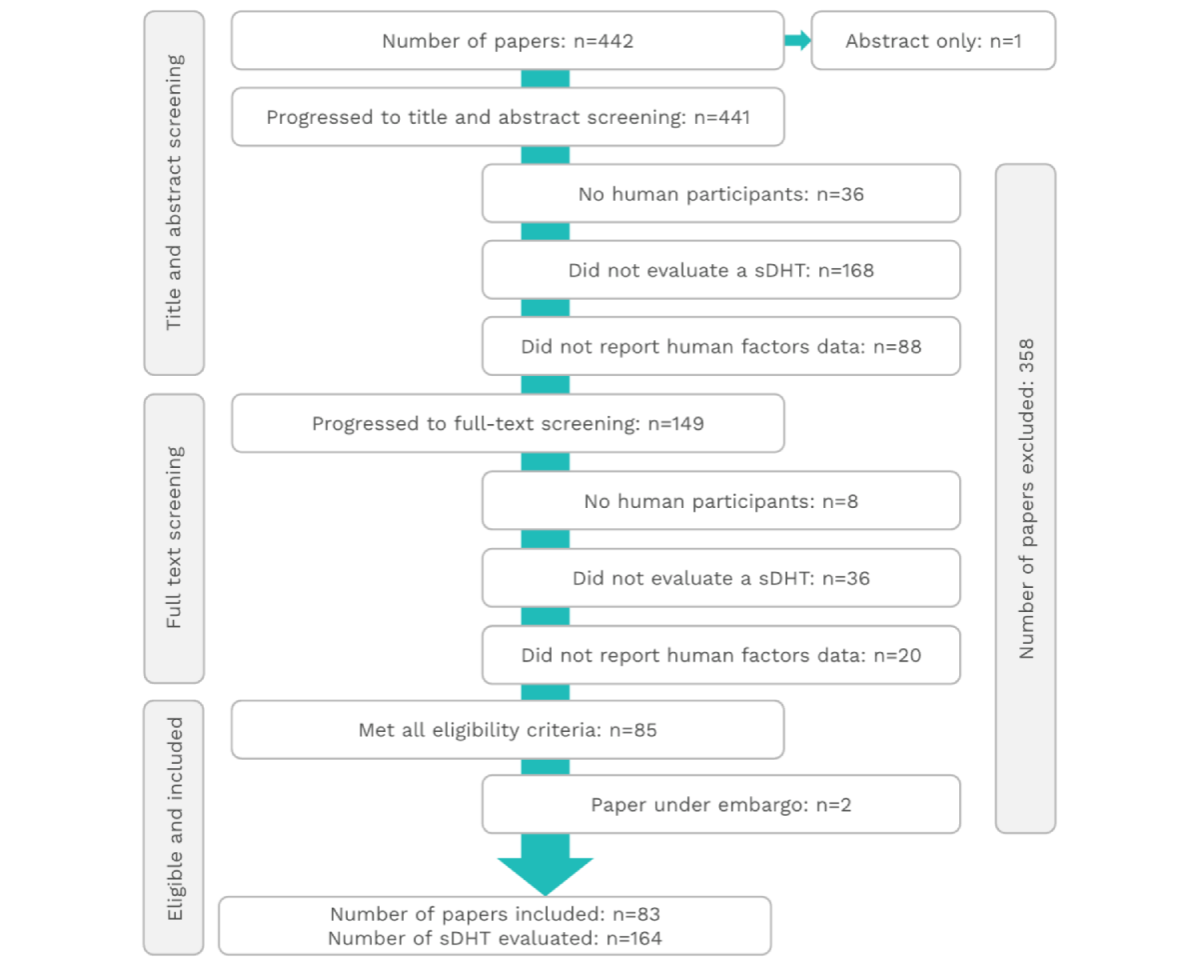
PRISMA flowchart. PRISMA: Preferred Reporting Items for Systematic Reviews and Meta-Analyses; sDHT: sensor-based digital health technology.

The PubMed search conducted on June 1, 2023, yielded 442 results, including one published only as an abstract. After applying the eligibility criteria described in Table 1, a further 356 publications were excluded. As such, 85 studies were determined to be eligible; however, 2 studies were under embargo, leaving 83 studies for data extraction (Figure 1). A complete list of all included studies is provided in Table S2 in Multimedia Appendix 2 [9,10,34-114].

As described above, two investigators reached a consensus on 20% (n=88) of the 442 publications, before any further publications were screened. The same investigators then screened a further 88 publications independently, which resulted in 100% agreement of eligibility. Per protocol, a single investigator screened the remaining 266 papers.

### Study Design Considerations

The majority of studies (55/83, 66%; Table 3) reported summative evaluations of products that were marketed or production-equivalent (ie, sample products of final design assembled in a way that differs from—but is equivalent to—the manufacturing processes used for the marketed product [115]). The remaining 28 (34%) out of 83 studies reported formative evaluations of prototype products; we did not identify any reports focused solely on sDHT design. Most studies (53/83, 64%) were conducted partially or completely off-site. Study sample sizes spanned a wide range (range 1-623; median 27, IQR 13-60); however, only 21 (25%) of the full set of 83 studies, and 17 (33%) of the 55 summative studies, reported a rationale for the sample size (with or without a power calculation).

**Table 3:** Study design and sample characteristics across therapeutic areas.

		Therapeutic area of sDHT^a^ end users (number of studies in parenthesis)									
		Aging (n=19)	Cardiovascular (n=9)	Endocrine (n=3)	Neurology (n=13)	Oncology (n=3)	Respiratory (n=6)	Surgery (n=5)	Healthy (n=15)	Other^b^ (n=10)	Total (n=83)
**Study design, n (%)**											
	Observational	17 (89)	9 (100)	3 (100)	13 (100)	3 (100)	5 (83)	4 (80)	14 (93)	10 (100)	78 (94)
	Interventional	2 (11)	0 (0)	0 (0)	0 (0)	0 (0)	1 (17)	1 (20)	1 (7)	0 (0)	5 (6)
**Study focus^c^** **, n (%)**											
	Summative; sample size rationale	3 (16)	3 (33)	0 (0)	4 (31)	0 (0)	1 (17)	2 (40)	1 (7)	3 (30)	17 (20)
	Summative; no sample size rationale	6 (32)	2 (22)	2 (67)	7 (54)	3 (100)	4 (67)	1 (20)	9 (60)	4 (40)	38 (46)
	Formative; sample size rationale	2 (11)	1 (11)	0 (0)	0 (0)	0 (0)	0 (0)	0 (0)	0 (0)	1 (10)	4 (5)
	Formative; no sample size rationale	8 (42)	3 (33)	1 (33)	2 (15)	0 (0)	1 (17)	2 (40)	5 (33)	2 (20)	24 (29)
**Setting, n (%)**											
	Remote	11 (58)	3 (33)	1 (33)	5 (38)	2 (67)	6 (100)	1 (20)	7 (47)	6 (60)	42 (51)
	On-site	4 (21)	6 (67)	1 (33)	5 (38)	0 (0)	0 (0)	3 (60)	8 (53)	3 (30)	30 (36)
	Both remote and on-site	4 (21)	0 (0)	1 (33)	3 (23)	1 (33)	0 (0)	1 (20)	0 (0)	1 (10)	11 (13)
**Duration of sDHT data collection, n (%)**											
	≤1 day	5 (26)	3 (33)	1 (33)	2 (15)	0 (0)	0 (0)	2 (40)	7 (47)	2 (20)	22 (27)
	>1 to ≤7 days	2 (11)	3 (33)	0 (0)	4 (31)	0 (0)	2 (33)	2 (40)	2 (13)	2 (20)	17 (20)
	>7 to ≤30 days	6 (32)	2 (22)	1 (33)	3 (23)	1 (33)	0 (0)	1 (20)	2 (13)	0 (0)	16 (19)
	>31 to ≤90 days	3 (16)	0 (0)	0 (0)	2 (15)	1 (33)	4 (67)	0 (0)	3 (20)	1 (10)	14 (17)
	>90 to ≤180 days	1 (5)	1 (11)	0 (0)	0 (0)	1 (33)	0 (0)	0 (0)	0 (0)	3 (30)	6 (7)
	>180 days	1 (5)	0 (0)	0 (0)	2 (15)	0 (0)	0 (0)	0 (0)	0 (0)	1 (10)	4 (5)
	Not reported	1 (5)	0 (0)	1 (33)	0 (0)	0 (0)	0 (0)	0 (0)	1 (7)	1 (10)	4 (5)
**Study sample**											
	Sample size, median (IQR)	30 (13.5-52.5)	24 (10-41)	35 (20-189)	40 (22-70)	30 (22-31.5)	14.5 (9.25-19.75)	29 (15-60)	25 (13-105)	21 (12-81.25)	27 (13-60)
	Sample size, range	8-125	5-156	5-343	5-623	14- 33	1-314	10-77	1-243	3-407	1-623
**Users, n (%)**											
	End users^d^	19 (100)	9 (100)	3 (100)	13 (100)	3 (100)	6 (100)	5 (100)	15 (100)	9 (90)	82 (99)
	Care partner users^d^	0 (0)	1 (11)	1 (33)	2 (15)	0 (0)	0 (0)	0 (0)	3 (20)	1 (10)	8 (10)
	Clinician users^d^	2 (11)	3 (33)	1 (33)	2 (15)	0 (0)	1 (17)	1 (20)	1 (7)	1 (10)	12 (14)
	Experts^d^	1 (5)	0 (0)	1 (33)	0 (0)	0 (0)	0 (0)	0 (0)	1 (7)	0 (0)	3 (4)
**Age, n (%)**											
	Adults only	19 (100)	7 (78)	1 (33)	10 (77)	2 (67)	3 (50)	5 (100)	11 (73)	7 (70)	65 (78)
	Children only	0 (0)	0 (0)	1 (33)	1 (8)	0 (0)	2 (33)	0 (0)	3 (20)	3 (30)	10 (12)
	Both adults and children	0 (0)	1 (11)	0 (0)	2 (15)	1 (33)	1 (17)	0 (0)	1 (7)	0 (0)	6 (7)
	Not reported	0 (0)	1 (11)	1 (33)	0 (0)	0 (0)	0 (0)	0 (0)	0 (0)	0 (0)	2 (2)
**Sex/gender, n (%)**											
	Male or men only	0 (0)	0 (0)	0 (0)	0 (0)	0 (0)	0 (0)	0 (0)	1 (7)	2 (20)	3 (4)
	Female or women only	1 (5)	0 (0)	0 (0)	0 (0)	1 (33)	1 (17)	0 (0)	0 (0)	2 (20)	5 (6)
	Both or all sexes/genders	18 (95)	7 (78)	2 (67)	13 (100)	2 (67)	5 (83)	5 (100)	13 (87)	6 (60)	71 (86)
	Not reported	0 (0)	2 (22)	1 (33)	0 (0)	0 (0)	0 (0)	0 (0)	1 (7)	0 (0)	4 (5)
**Race/ethnicity, n (%)**											
	Race/ethnicity reported	2 (11)	1 (11)	0 (0)	4 (31)	0 (0)	2 (33)	0 (0)	3 (20)	2 (20)	14 (17)
	Race/ethnicity not reported	17 (89)	8 (89)	3 (100)	9 (69)	3 (100)	4 (67)	5 (100)	12 (80)	8 (80)	69 (83)
**Number of sDHTs assessed**											
	Range	1-7	1-3	1-1	1-5	1-6	1-5	1-2	1-7	1-11	1-11

^a^sDHT: sensor-based digital health technology.

^b^“Other” therapeutic area category contains studies with enrollment eligibility focused on anaphylaxis, muscular dystrophy, hemophilia, nocturnal enuresis, blood and marrow transplant, overweight or obesity, pregnancy, and nonspecific hospitalized or chronic illness. One study recruited clinicians only (no end users of the sDHT) and is included in this category.

^c^Studies reporting formative and summative evaluations are categorized as summative.

^d^Categories are not mutually exclusive.

### Sample Characteristics

As shown in Table 3, the largest target populations were focused on aging and healthy participants (15 and 19 studies, respectively; 34/83, 41% of all studies). Among the various diseases studied, neurology and cardiovascular were the most common therapeutic areas (13 and 9 studies, respectively; 22/44, 50% of studies assessing nonhealthy individuals). Table S3 in Multimedia Appendix 2 contains a list of conditions falling into each therapeutic area.

Almost all studies (82/83, 99%) captured data from targeted end users; the remaining study captured data only from clinician users [63]. Several studies captured data from multiple user groups; in total, 8 and 12 studies gathered data from care partner users and clinician users, respectively. Three studies involved experts (not considered to be sDHT users); two of these described a formal heuristic evaluation [79,99] while the other described involving experts in design, biomedical engineering, computer science, and mobile health system production in the sDHTs design and formative testing process [103]. Finally, we noted substantial missing participant demographic data; age, sex/gender, and race/ethnicity were not reported in 2, 4, and 69 studies, respectively.

### sDHTs Assessed in Eligible Studies

Across the 83 studies included in our review, a total of 164 different sDHTs were assessed (141 wearable and 23 ambient tools; Table 4), ranging from 1 to 11 sDHTs within a single study. Ingestible and implantable sDHTs were in scope, but none were identified in our literature search. A wide range of form factors (22 distinct categories) and wear locations (14 anatomical locations presented in 5 categories) were identified. Digital clinical measures of vital signs (n=76 sDHTs), physical activity (n=61 sDHTs), and mobility (n=35) were most prevalent. Table S4 in Multimedia Appendix 2 contains more comprehensive information regarding wear locations and health concepts captured by sDHTs.

Most sDHTs (126/164, 77%) required only passive interaction by users, meaning that data were captured without user input other than basic tasks such as charging or changing batteries. The remaining 38 (23%) sDHTs required active engagement at specific times such as completion of physical therapy [58], exercise [97], or blood glucose tests [79].

**Table 4:** sDHT^a^ descriptive information across therapeutic areas.

		Therapeutic area of sDHT users (number of sDHTs in parenthesis)									
		Aging (n=27)	Cardiovascular (n=12)	Endocrine (n=3)	Neurology (n=31)	Oncology (n=8)	Respiratory (n=15)	Surgery (n=7)	Healthy (n=35)	Other^b^ (n=26)	Total (n=164)
**sDHT type, n (%)**											
	Wearable	26 (96)	9 (75)	1 (33)	30 (97)	8 (100)	11 (73)	7 (100)	33 (94)	16 (62)	141 (86)
	Ambient	1 (4)	3 (25)	2 (67)	1 (3)	0 (0)	4 (27)	0 (0)	2 (6)	10 (38)	23 (14)
**sDHT maturity, n (%)**											
	Prototype	9 (33)	5 (42)	1 (33)	5 (16)	0 (0)	0 (0)	2 (29)	5 (14)	11 (42)	38 (23)
	Final or marketed	18 (67)	6 (50)	2 (67)	26 (84)	8 (100)	15 (100)	5 (71)	27 (77)	12 (46)	119 (73)
	Not reported	0 (0)	1 (8)	0 (0)	0 (0)	0 (0)	0 (0)	0 (0)	3 (9)	3 (12)	7 (4)
**Form factor, n (%)**											
	Adhesive patch	2 (7)	0 (0)	1 (33)	5 (16)	0 (0)	0 (0)	1 (14)	2 (6)	1 (4)	12 (7)
	Balance board	0 (0)	0 (0)	0 (0)	0 (0)	0 (0)	0 (0)	0 (0)	0 (0)	1 (4)	1 (<1)
	Camera, video, or still	0 (0)	1 (8)	0 (0)	0 (0)	0 (0)	0 (0)	0 (0)	0 (0)	2 (8)	3 (2)
	Clip	4 (15)	0 (0)	0 (0)	0 (0)	0 (0)	1 (7)	1 (14)	1 (3)	0 (0)	7 (4)
	Clothing or shoes	5 (19)	3 (25)	0 (0)	0 (0)	0 (0)	0 (0)	0 (0)	4 (11)	5 (19)	17 (10)
	Contact lens	0 (0)	0 (0)	0 (0)	1 (3)	0 (0)	0 (0)	0 (0)	0 (0)	0 (0)	1 (<1)
	Cuff or wrap	0 (0)	1 (8)	0 (0)	1 (3)	0 (0)	0 (0)	1 (14)	2 (6)	0 (0)	5 (3)
	Electrode or electrodes	0 (0)	0 (0)	0 (0)	1 (3)	0 (0)	0 (0)	0 (0)	2 (6)	0 (0)	3 (2)
	Exercise equipment	0 (0)	0 (0)	1 (33)	0 (0)	0 (0)	0 (0)	0 (0)	0 (0)	2 (8)	3 (2)
	Glasses	1 (4)	0 (0)	0 (0)	0 (0)	0 (0)	0 (0)	0 (0)	1 (3)	0 (0)	2 (<1)
	Gloves	2 (7)	1 (8)	0 (0)	1 (3)	0 (0)	0 (0)	0 (0)	0 (0)	0 (0)	4 (2)
	Glucometer	0 (0)	0 (0)	1 (33)	0 (0)	0 (0)	0 (0)	0 (0)	0 (0)	0 (0)	1 (<1)
	Handheld thermometer	0 (0)	0 (0)	0 (0)	0 (0)	0 (0)	1 (7)	0 (0)	0 (0)	0 (0)	1 (<1)
	Mattress pad	0 (0)	1 (8)	0 (0)	1 (3)	0 (0)	0 (0)	0 (0)	0 (0)	0 (0)	2 (<1)
	Medication package	0 (0)	0 (0)	0 (0)	0 (0)	0 (0)	1 (7)	0 (0)	0 (0)	1 (4)	2 (<1)
	Phone or tablet	0 (0)	0 (0)	0 (0)	0 (0)	0 (0)	0 (0)	0 (0)	1 (3)	4 (15)	5 (3)
	Probe	0 (0)	0 (0)	0 (0)	1 (3)	0 (0)	0 (0)	0 (0)	0 (0)	1 (4)	2 (<1)
	Ring	0 (0)	0 (0)	0 (0)	0 (0)	0 (0)	1 (7)	0 (0)	0 (0)	0 (0)	1 (<1)
	Spirometer	0 (0)	0 (0)	0 (0)	0 (0)	0 (0)	2 (13)	0 (0)	0 (0)	0 (0)	2 (<1)
	Contactless unit	1 (4)	0 (0)	0 (0)	0 (0)	0 (0)	0 (0)	0 (0)	1 (3)	0 (0)	2 (<1)
	Strap	12 (44)	4 (33)	0 (0)	20 (65)	8 (100)	9 (60)	4 (57)	21 (60)	9 (35)	87 (53)
	Weight scale	0 (0)	1 (8)	0 (0)	0 (0)	0 (0)	0 (0)	0 (0)	0 (0)	0 (0)	1 (<1)
**Wear location, n (%)**											
	Arms or wrists or hands	11 (41)	5 (42)	0	19 (61)	8 (100)	8 (53)	3 (43)	19 (54)	9 (35)	82 (50)
	Head or face	1 (4)	0	0	4 (13)	0	0	0	3 (9)	0	8 (5)
	Legs or ankles or feet	2 (7)	2 (17)	0 (0)	1 (3)	0 (0)	0 (0)	2 (29)	3 (9)	0 (0)	10 (6)
	Neck or torso or hips	10 (37)	2 (17)	1 (33)	3 (10)	0 (0)	2 (13)	2 (29)	6 (17)	6 (23)	32 (20)
	Multiple locations^c^	2 (7)	0 (0)	0 (0)	3 (10)	0 (0)	0 (0)	0 (0)	2 (6)	1 (4)	8 (5)
	N/A^d^	1 (4)	3 (25)	2 (67)	1 (3)	0 (0)	5 (33)	0 (0)	2 (6)	10 (38)	24 (15)
**Interaction type^e^** **, n (%)**											
	Passive	24 (89)	7 (58)	1 (33)	26 (84)	8 (100)	12 (80)	3 (43)	30 (86)	15 (58)	126 (77)
	Active	3 (11)	5 (42)	2 (67)	5 (16)	0 (0)	3 (20)	4 (57)	5 (14)	11 (42)	38 (23)
**Health concepts^f^**, **n (%)**											
	Activities of daily living	6 (22)	2 (17)	0 (0)	2 (6)	0 (0)	0 (0)	0 (0)	1 (3)	0 (0)	11 (7)
	Physical activity	16 (59)	4 (33)	1 (33)	6 (19)	9 (113)	9 (6)	1 (14)	15 (43)	0 (0)	61 (37)
	Adherence	0 (0)	0 (0)	0 (0)	0 (0)	0 (0)	1 (7)	0 (0)	0 (0)	0 (0)	1 (<1)
	Electrical activity	0 (0)	0 (0)	0 (0)	15 (48)	0 (0)	0 (0)	0 (0)	1 (3)	0 (0)	16 (<1)
	Mobility	5 (19)	4 (33)	0 (0)	11 (35)	0 (0)	0 (0)	2 (29)	13 (37)	0 (0)	35 (21)
	Sleep	6 (22)	1 (8)	0 (0)	0 (0)	1 (13)	1 (7)	0 (0)	5 (14)	0 (0)	14 (9)
	Vital signs	9 (33)	5 (42)	2 (67)	18 (58)	0 (0)	5 (33)	14 (2)	23 (66)	0 (0)	76 (46)
	Other^g^	4 (15)	2 (17)	0 (0)	4 (13)	0 (0)	2 (13)	0 (0)	0 (0)	0 (0)	12 (7)

^a^sDHT: sensor-based digital health technology.

^b^“Other” therapeutic area category contains studies with enrollment eligibility focused on anaphylaxis, muscular dystrophy, hemophilia, nocturnal enuresis, blood and marrow transplant, overweight or obesity, pregnancy, and nonspecific hospitalized or chronic illness. One study recruited clinicians only (no end users of the sDHT) and is included in this category.

^c^Refers to multisensor sDHTs worn on different parts of the body, or sDHTs that can be positioned in one of many locations.

^d^Wear location is not applicable to ambient sDHTs. Wear locations are presented in greater detail in Table S4 in Multimedia Appendix 2.

^e^Passive: sDHT data are collected over long time periods without user input other than aspects such as charging or changing batteries (such as actigraphy); includes tools for which the absence of data is meaningful (such as smart packaging for adherence monitoring). Active: sDHT data collection requires user engagement at defined timepoints. Categories described previously [28].

^f^Health concepts are not mutually exclusive; a single sDHT can capture data in multiple categories. Heath concepts are presented in greater detail in Table S4 in Multimedia Appendix 2.

^g^“Other” health concept category includes bladder volume, body habitus, cardiac output, fall detection, gaze or visual movement, intraocular pressure, lung or airway function, and tremor detection.

### Methodological Approaches

As described in Table 5, most sDHTs (139/164, 85%) were evaluated in the actual environment in which they were intended to be used, while 25 sDHTs were assessed in a simulated environment only. The vast majority were evaluated during actual use (148/164, 90%) rather than through “look and feel” approaches. Of particular interest, a variety of methods were used to evaluate usability and related concepts, including interviews (49 sDHTs), focus groups (29 sDHTs), direct or video observation (35 sDHTs), think-aloud (15 sDHTs), and heuristic analysis (2 sDHTs). Surveys were the most prevalent method for capturing usability data; 86 sDHTs were evaluated using referenced surveys while 81 sDHTs were evaluated using surveys developed in house by study investigators. Data for 4 sDHTs were captured using the sDHT itself; for example, instances of connectivity loss or data capture drops were recorded as “use errors” or “technical performance or product errors” [42,100]. An illustrative example of a use error that may be addressed through design modification is the report of users turning an sDHT on and off repeatedly as it was not clear whether the product was operating correctly [53]. Additional examples of product errors, distinct from use errors, included instances of system crash [51] and software malfunctions requiring computer program patches [54].

**Table 5:** Methodological approaches to usability data collection.

			sDHT^a^ type (number of sDHTs in parenthesis)				
			Ambient (n=23)		Wearable (n=141)		Total (n=164)
**Data collection environment, n (%)**							
	Actual environment	23 (100)		107 (76)		130 (79)	
	Simulated environment	0 (0)		25 (18)		25 (15)	
	Both actual and simulated	0 (0)		9 (6)		9 (5)	
**Interactions with sDHT, n (%)**							
	Look and feel	1 (4)		15 (11)		16 (10)	
	Actual use	22 (96)		126 (89)		148 (90)	
**Usability evaluation methods^b^**							
	Interviews	5 (22)		44 (31)		49 (30)	
	Focus groups	10 (43)		19 (13)		29 (18)	
	Surveys—referenced	14 (61)		72 (51)		86 (52)	
	Surveys—in house	7 (30)		74 (52)		81 (49)	
	Think-aloud	1 (4)		14 (10)		15 (9)	
	Observation (direct or video)	1 (4)		34 (24)		35 (21)	
	Measured by the sDHT	0 (0)		5 (4)		5 (3)	
	Heuristic analysis	1 (4)		1 (<1)		2 (<1)	
**Type or types of usability data reported, n (%)**							
	Mixed methods	14 (61)		58 (41)		72 (44)	
	Quantitative only	6 (26)		60 (43)		66 (40)	
	Qualitative only	3 (13)		23 (16)		26 (16)	
**Categories of usability and related data reported, n (%)**							
	User satisfaction	19 (83)		117 (83)		136 (83)	
	Comfort	5 (22)		107 (76)		112 (68)	
	Ease of use; self-report	23 (100)		122 (87)		145 (88)	
	Ease of use; objectively captured	1 (4)		4 (3)		5 (3)	
	Learnability	1 (4)		10 (7)		11 (7)	
	Efficiency	0 (0)		4 (3)		4 (2)	
	Memorability	0 (0)		2 (<1)		2 (<1)	
	Usefulness	16 (70)		96 (68)		112 (68)	
	Use errors	6 (26)		26 (18)		32 (20)	
	User trust	12 (52)		53 (38)		65 (40)	
	Readability	0 (0)		0 (0)		0 (0)	
	Understandability or actionability	1 (4)		13 (9)		14 (9)	
	Technical performance or product errors	19 (83)		79 (56)		98 (60)	
**Adherence to sDHT reported, n (%)**							
	Objectively measured by the sDHT	6 (26)		44 (31)		50 (30)	
	Self or care partner report	1 (4)		14 (10)		15 (9)	
	Both objective and self or care partner	0 (0)		4 (3)		4 (2)	
	Reported but method not described	1 (4)		7 (5)		8 (5)	
	Adherence not reported	15 (65)		72 (51)		87 (53)	

^a^sDHT: sensor-based digital health technology.

^b^Categories are not mutually exclusive.

### Categories of Usability-Related Data Reported

User satisfaction was captured for the majority of sDHTs (136/164, 83%), often as a measure of acceptability or user attitudes. Although overall ease of use was also commonly reported, captured through either self-report or objective methods (n=145 and n=5, respectively), the related concepts of learnability, efficiency, and memorability were reported for only 11, 4, and 2 sDHTs, respectively. Technical performance and product errors associated with malfunction were captured for 98 sDHTs, while use errors were captured for only 32 sDHTs. Finally, although none of the studies in our review reported the readability of information presented to the user, 14 sDHTs were evaluated according to the extent to which users were able to understand the data or information presented to them (understandability) or the actions or tasks they should complete in response (actionability).

Finally, adherence (such as wear or use time) was reported for 77 sDHTs. Of these, 50 sDHTs captured adherence data objectively, adherence to 19 sDHTs was assessed through self-report or care partner report, and the method was not described for 8 sDHTs.

**Figure 2:**
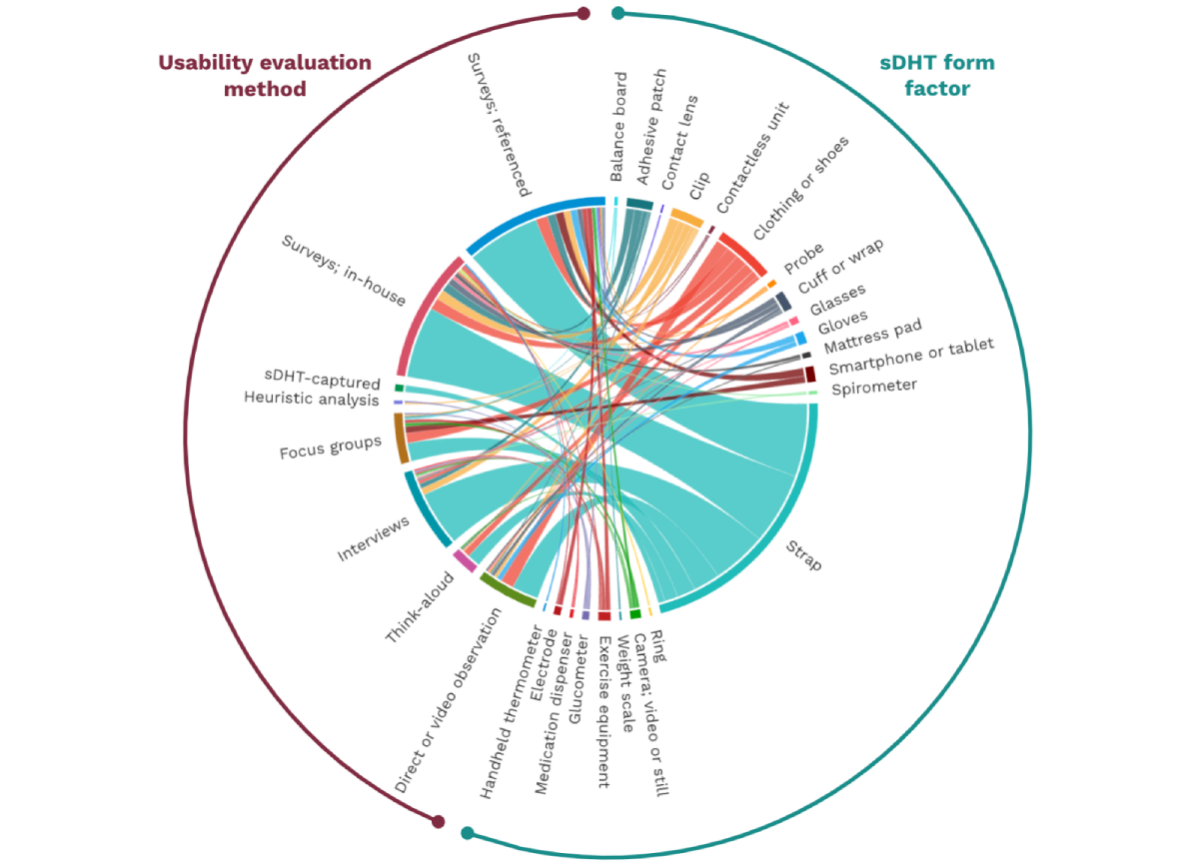
Chord diagram depicting the relationship between sDHT form factors and usability evaluation methods. sDHT: sensor-based digital health technology.

**Figure 3:**
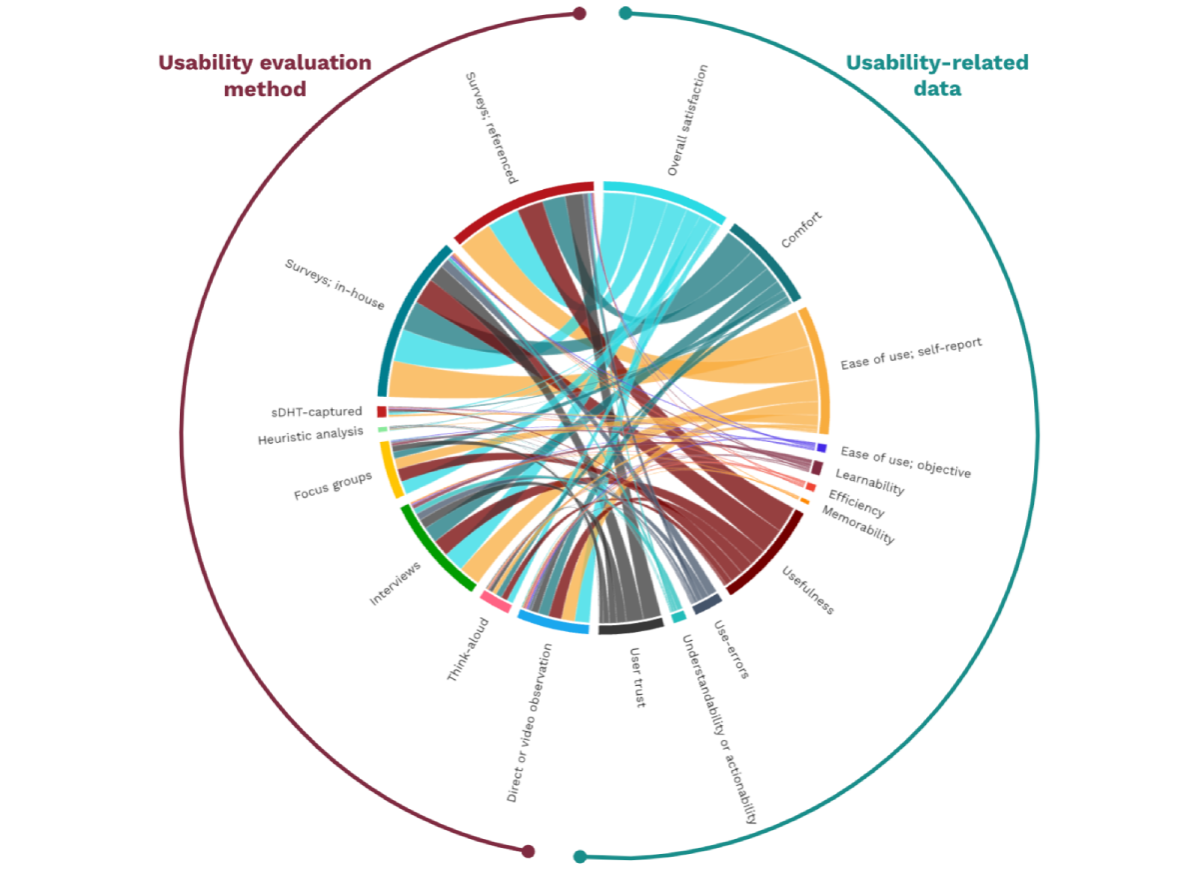
Chord diagram depicting the relationship between usability evaluation methods and categories of usability-related data reported. sDHT: sensor-based digital health technology.

The complexity of the relationships in our dataset comparing usability evaluation methods with sDHT form factor, and comparing usability evaluation methods with the categories of usability-related data reported, are depicted in Figures 2 and 3, respectively. For example, the width of each chord in Figure 2 is proportional to the number of sDHTs of the relevant form factor that were assessed using the linked method, demonstrating that surveys (both referenced and in house) were the most common evaluation methods while heuristic analysis was the least common. Similarly, Figure 3 demonstrates that overall satisfaction and self-reported ease of use were captured frequently, in contrast to data related to objective ease of use, efficiency, learnability, and memorability. Both figures contain a large number of linked chords, indicating that specific usability evaluation methods were adopted across diverse sDHT form factors and outcome measures.

Tables S5-S7 in Multimedia Appendix 2 present the data shown in Tables 3-5 for the subset of 55 studies reporting the results of summative evaluations, while Tables S8-S10 in Multimedia Appendix 2 present these data for the subset of 28 studies reporting formative evaluations.

## Discussion

### Principal Findings

This paper represents the first scoping review reporting the methodological approaches adopted during usability-related studies specifically focused on sDHTs. We identified 83 formative and summative studies published over the decade from 2013 to 2023 that evaluated human factors, human-centered design, or usability for 164 ambient and wearable tools. Most studies (67/83, 81%) recruited nonhealthy individuals, thereby providing informative data regarding sDHT usability across many diseases in addition to other aspects of health such as aging and pregnancy. Most sDHTs were evaluated in the intended use environment, with multiple facets of usability-related data captured via a range of mixed method approaches including heuristic analysis, surveys, observation, think-aloud, focus groups, interviews, and use errors or technical performance errors captured by the sDHT itself such as instances of connectivity loss.

This review highlights 4 notable gaps that warrant attention as the field advances. First, the breadth and scope of usability and related data were fairly simplistic, relying largely on surveys capturing user satisfaction and ease of use (each captured for >80% of sDHTs) with limited reporting of sDHT use errors, learnability, efficiency, or memorability. The extent to which users understood the health- and behavior-related data or other information presented to them (understandability) and the actions or tasks they should complete in response (actionability) was assessed for only 9% (14/164) of sDHTs. Understandability and actionability are particularly important for sDHTs, given that they are often used by patients or participants in out-of-clinic settings without clinical supervision. For the use of sDHTs in clinical care settings, it is imperative that users understand whether and how to react to clinical data [116], and thus the lack of focus on understandability and actionability is concerning and could be due to the early-stage nature of sDHTs in clinical practice. In the context of clinical research, however, sharing sDHT data with participants in real time has the potential to introduce bias and affect user behavior, thereby posing a risk of yielding inaccurate results [4]. Additional dimensions related to understandability and actionability, such as understanding optimal ways of implementing remote examinations, also warrant further investigation.

Second, only 22% (18/83) of studies considered users other than end users (patients or participants), such as care partners and clinicians, who play crucial roles in sDHT implementation and therefore the quality of data captured [117]. Especially in populations where care partners play a key role in sDHT implementation (eg, children, older people, those with language barriers, and those with disabilities), understanding usability from the care partner perspective is vital. Although existing usability data may be available for some sDHTs that are regulated as medical devices, research participants likely have needs and motivations for using the sDHT that differ from patients using the product as part of usual care. Similarly, the needs of investigator users are likely different from the needs of clinician users, requiring further evaluation.

Third, we found that only 31% (17/55) of summative studies (referred to by the US Food and Drug Administration as “human factors validation studies”; [20]) provided a rationale for the sample size, with or without a power calculation. An understanding of key study design considerations, including sample size, is important for evaluating the robustness of study conclusions.

Finally, as has been noted previously [28,118], we observed a deficiency in reporting basic sample demographics, with studies typically providing information on age and sex/gender but neglecting to include details on the race and ethnicity of participants. Inadequate reporting of descriptive data, including sociodemographics, precludes a complete understanding of generalizability, potentially leading to the need to repeat studies while contributing to disparities and biases in clinical research [119].

As described above, while there are several existing systematic reviews describing the usability of digital health products for specific applications [15-19], few have focused specifically on evaluating methodological approaches. In addition, most prior systematic reviews with similar objectives have focused on digital health technologies that are not sensor-based, such as electronic medical records systems [120] and mobile clinical decision support tools [121], that are not used for remote data capture. In 2023, Maqbool and Herold [5] published a systematic review of usability evaluations describing a broad suite of over 1000 digital health tools consisting mostly of mobile health apps and including a subset of 20 products approximately aligned to our definition of sDHT, including fitness or activity trackers, digital sphygmomanometers, and wearable fall risk assessment systems. Compared to this study, Maqbool and Herold [5] found relatively increased rates of clinician and care partner participation, and reporting of learnability, efficiency, and memorability. Such differences emphasize substantial variability in usability study methodology across subcategories of digital health technologies, as well as differences in definitions and terminology of the concepts reported, underscoring the need for a common evaluation framework.

### Strengths and Limitations

Strengths of this review include the robust approach taken to testing our search terms including a careful assessment against a list of target papers identified a priori to ensure that we were capturing appropriate literature. This process was intended to not only ensure the inclusivity of relevant literature but also the reliability of our findings to help provide a foundation for subsequent reviews and meta-analyses. In-depth data extraction across many domains allowed for a thorough comparative analysis of the identified studies. The decision to focus on studies published within the last decade (2013-2023) was also carefully considered, as it encompasses the recent surge in studies reporting sDHT implementation. While sDHTs have a lengthy history prior to 2013, this temporal scope ensures that our findings reflect contemporary developments and trends, offering insights into the current state of sDHT implementation.

A number of limitations are acknowledged. First, we limited our search to the peer-reviewed literature. We acknowledge that many usability studies undertaken by technology manufacturers may be published in the gray literature; however, our ultimate goal is to use the findings of our review to guide the development of a framework representing best practices, and therefore, the peer review process was used as an indicator of methodological rigor and reporting quality. Second, terminology in the field of digital medicine is still evolving and investigators use many different terms to describe sDHTs; by incorporating 25 descriptive keywords in Layer C of our search terms (Table S1 in Multimedia Appendix 2), we found it necessary to rely on MeSH terms developed by the National Library of Medicine [26] as a means of limiting our literature search to a feasible number of publications. As a consequence, we were limited to conducting our search in PubMed as this is the clinical research database for the National Library of Medicine. While MeSH terms are widely accepted and systematically applied, their specificity may have excluded relevant studies using different terminology potentially resulting in unintentional omissions. In addition, the decision to search within one database may have resulted in missed publications. Our hope is that as the field matures, terminology will become harmonized and sDHT-specific indexing will support the identification of studies adopting these technologies. Third, our decision to exclude descriptions of specific sensors, form factors, methodologies, wear location, and technology make or model may have excluded publications that used these types of keywords in the absence of other descriptors and MeSH terms. This approach was taken to reduce the possibility of overlooking novel or emerging technologies in favor of established digital products such as actigraphy tools. Finally, only 40% (176/442) of publications were screened for eligibility by multiple investigators. This approach to study identification, which has been described and adopted previously [27,28], allowed us to screen a greater number of papers which was necessary given the lack of systematic indexing. The high agreement levels between investigators suggest that our quality-control approach maintained a robust screening process, despite part of the work being conducted by a single investigator.

### Conclusions and Future Directions

Based on our findings, we suggest 4 actionable recommendations that will help to advance the implementation of sensor-based digital measurement tools in both clinical and research settings. First, we encourage investigators to adopt in-depth assessment and reporting of usability data beyond user satisfaction and ease of use. In particular, it is valuable to understand use errors alongside technical errors, and it is critical to evaluate the extent to which users understand the clinical data and information presented to them and the appropriate tasks to undertake in response, if applicable. Second, it is essential to embrace the diversity of users in all respects, including diversity of stakeholders within the human-centered design process; evaluation of usability across multiple user groups including care partners and clinicians; and ensuring that the participating users are generalizable to the intended use population in terms of sociodemographics, social determinants of health, and other characteristics. Third, rigorous study design is key. Usability is a heterogeneous concept, and it is often beneficial to evaluate usability alongside other objectives such as analytical or clinical validation; thus, we do not advocate a particular study design or set of study outcome measures. We do, however, believe that careful consideration of usability evaluation criteria, study sample sizes, and predetermined thresholds of success is critical for making go or no-go decisions as to whether a particular sDHT is sufficiently usable for implementation in a particular context of use. Finally, we recommend adhering to reporting and publication checklists such as Annex B in ISO 9241-11:2018 [122] and EVIDENCE [123], the latter of which describes optimal reporting requirements of studies evaluating several aspects of sDHT quality including usability assessments. Ensuring consistency in reporting will enable meaningful comparisons between studies, facilitate better assessments of findings, and enhance the accurate interpretation of results and limitations across studies.

Our long-term goal is to develop and disseminate an evidence-driven framework for evaluating sDHTs as being fit for purpose from a usability perspective, informed in part by the findings of this review. By developing such a framework, we endeavor to contribute to the ongoing discourse surrounding sDHTs, ultimately paving the way for the development of safe and effective tools that lead to a more inclusive and patient-centric health care ecosystem poised to improve clinical trials and clinical practice.

## Appendix

Multimedia Appendix 1 PRISMA-ScR (Preferred Reporting Items for Systematic Reviews and Meta-Analyses Extension for Scoping Reviews) checklist.

Multimedia Appendix 2 Supplementary tables.

## Abbreviations

MeSH: Medical Subject Heading
PICO: patients/participants; intervention; comparator; outcomes
PRISMA: Preferred Reporting Items for Systematic Reviews and Meta-Analyses
sDHT: sensor-based digital health technology
